# Large Ovarian Fibroma With Torsion in a 17-Year-Old Patient: A Report of a Rare Case of Acute Surgical Abdomen

**DOI:** 10.7759/cureus.96141

**Published:** 2025-11-05

**Authors:** Emmanouil M Xydias, Anna Thanasa, Efthymia Thanasa, Vasiliki Koutsia, Ioannis Tsartsalis, Evangelos Kamaretsos, Apostolos C Ziogas, Ioannis Thanasas

**Affiliations:** 1 Department of Obstetrics and Gynecology, EmbryoClinic IVF, Thessaloniki, GRC; 2 Department of Health Sciences, Medical School, Aristotle University of Thessaloniki, Thessaloniki, GRC; 3 Department of Obstetrics and Gynecology, General Hospital of Trikala, Trikala, GRC; 4 Department of Surgery, General Hospital of Trikala, Trikala, GRC; 5 Third Department of Obstetrics and Gynecology, University General Hospital "Attikon" Medical School, National and Kapodistrian University of Athens, Trikala, GRC; 6 Department of Obstetrics and Gynecology, University of Thessaly, Larissa, GRC

**Keywords:** case report, computed tomography, histological diagnosis, laparotomy, ovarian fibroma, prognosis, torsion

## Abstract

This study presents a rare case of emergency surgical management via exploratory laparotomy of a large torsed ovarian fibroma in a young female patient. A 17-year-old patient with no history of sexual activity and under chronic antipsychotic medication presented to the emergency department with signs of acute surgical abdomen and fever up to 37.9°C. Laboratory markers of inflammation were elevated. An emergency computed tomography scan revealed a large, solid echogenic pelvic mass of unclear origin. The clinical and laboratory findings, along with the imaging results, led to the decision to perform an emergency exploratory laparotomy. Intraoperatively, a large, solid ovarian mass in torsion, along with the ipsilateral fallopian tube, was identified. A right salpingo-oophorectomy was performed. Histological examination confirmed the diagnosis of a torsed ovarian fibroma. The immediate postoperative course was uneventful, and the patient was discharged on the fourth postoperative day. This article also includes a brief narrative review of the literature regarding ovarian fibromas with torsion, focusing on their rarity, diagnostic challenges, management, and prognosis.

## Introduction

Ovarian fibromas were first described as a distinct pathological entity by Young and Scully in 1983 [1]. However, as early as 1954, Joe Vincent Meigs had included ovarian fibroma along with the presence of ascites and pleural effusion among the official diagnostic criteria for Meigs syndrome [2]. Ovarian fibromas are benign stromal tumors of the ovary, typically occurring in perimenopausal and postmenopausal women, with a mean age of onset between the fifth and sixth decades of life [3]. The fibroma is the most common solid tumor of the ovary. The incidence of ovarian fibromas and fibrothecomas is estimated to be approximately 3.3% of all ovarian tumors [4].

In the majority of cases, ovarian fibromas are unilateral, non-functional tumors that do not produce hormones. Nevertheless, isolated cases have been reported in the literature of patients with ovarian fibroma in which the synthesis and production of steroid hormones were attributed to the tumor [3,5]. The size of ovarian fibromas varies from a few centimeters to large-sized ovarian fibromas. In a retrospective study, the average size of ovarian fibromas was found to be 14 cm [6]. It has been known for decades that, in rare cases, particularly in patients harboring large ovarian fibromas, torsion of the corresponding ovary may occur. Ovarian torsion, defined as a partial or complete rotation of the vascular pedicle of the corresponding adnexa, can cause obstruction of blood flow and lead to ischemia and necrosis of the ovarian tissue, for the effective management of which urgent surgical intervention is required [7].

This article presents a rare case of a 17-year-old female patient with acute abdominal symptoms. Imaging revealed a large pelvic mass, which was managed with emergency laparotomy. Intraoperative findings confirmed the adnexal origin of the torsed mass. Histological analysis confirmed the diagnosis of a torsed ovarian fibroma. A brief descriptive literature review focusing on the rarity, diagnosis, treatment, and prognosis of ovarian fibromas with torsion is also included.

## Case presentation

A 17-year-old female patient presented to the Emergency Department of the General Hospital of Trikala, Greece, reporting the sudden onset of severe right lower abdominal pain radiating to the right iliac fossa, beginning approximately 12 hours prior. The pain was unrelieved by oral paracetamol (Depon®), 1 g initially and another dose four hours later. She reported nausea and multiple episodes of vomiting about four hours after symptom onset. The patient denied any history of sexual intercourse. Her medical history was significant for chronic antipsychotic medication use. Her menstrual cycles were irregular with long periods of secondary amenorrhea. She had not previously consulted a gynecologist. One year earlier, she had undergone an appendectomy for acute appendicitis through an oblique McBurney incision. Her body mass index (BMI) was 34.5 (165 cm/94 kg), classifying her as obese.

**Figure 1:**
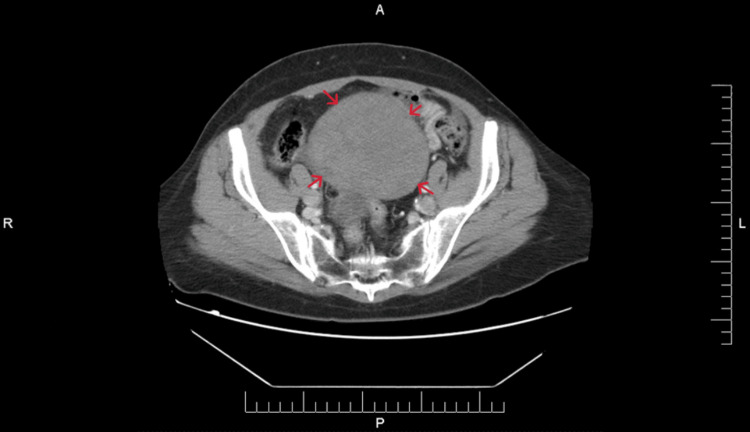
CT scan image of an ovarian fibroma with torsion A well-defined solid echogenic mass (red arrows) is clearly delineated in the pelvic cavity, most likely originating from the right ovary

On clinical examination, her temperature was 37.9°C, blood pressure was 113/72 mmHg, and pulse rate was elevated at 105 bpm. Abdominal palpation revealed diffuse tenderness, with rebound tenderness in the right lower quadrant extending from the lower pole of the right kidney to the right iliac fossa. Due to increased abdominal wall thickness, the transabdominal ultrasound provided limited information regarding the reproductive organs. A transvaginal ultrasound could not be performed. Renal, ureteric, and bladder ultrasonography showed no hydronephrosis. A computed tomography (CT) scan revealed a large solid echogenic pelvic mass measuring 18.9 x 17.4 cm, but its exact origin-ovarian or uterine-was unclear (Figure 1).

Laboratory results showed elevated inflammatory markers (Table 1). Urinalysis was unremarkable.

**Table 1:** Laboratory tests of the patient during her hospitalization at the obstetrics and gynecology department Ht, hematocrit; Hb, hemoglobin; PLT, platelets; WBC, white blood cellc; NEUT, neutrophils; CRP, C-reactive protein; APTT, activated partial thromboplastin time; INR, International Normalized Ratio; Glu, glucose; Cr, creatinine; K+, potassium; Na+, sodium; TBIL, total bilirubin; SGOT, serum glutamic oxaloacetic transaminass; SGPT, serum glutamate pyruvate transaminass; AMY, amylase

Laboratory tests	Preoperative	1st postoperative day	3rd postoperative day	Normal laboratory values
Ht	37.4%	33.7%	32.1%	37.7 – 49.7%
Hb	12.3 gr/dl	11.2 gr/dl	10.8 gr/dl	11.8 – 17.8 gr/dl
WBC	21.19x10^3^/ml	18.31x10^3^/ml	8.65x10^3^/ml	4 – 10.8 x10^3^/ml
NEUT	91.1%	87.1%	71.1%	40 – 75%
CRP	18.15 mg/dl	17.67 mg/dl	2.15 mg/dl	0.5 mg/dl
APPT	31.6	30.1	28.4	24.0 – 35.0 sec
INR	1.14	1.01	0.92	0.8 – 1.2
Glu	138 mg/dl	87 mg/dl	-	75 – 115 mg/dl
Cr	0.75 mg/dl	0.61 mg/dl	-	0.40 – 1.10 mg/dl
Na^+^	135.1 mmol/L	139.2 mmol/L	-	136 – 145 mmol/L
K^+^	4.31 mmol/L	4.01 mmol/L	-	3.5 – 5.1 mmol/L
TBIL	0.78 mg/dl	0.81 mg/dl	-	0 – 1.2 mg/dl
SGOT	19 IU/L	17 IU/L	-	5 – 33 IU/L
SGPT	15 IU/L	13 IU/L	-	10 – 37 IU/L
AMY	35 IU/L	31 IU/L	-	30 – 118 IU/L

**Figure 2:**
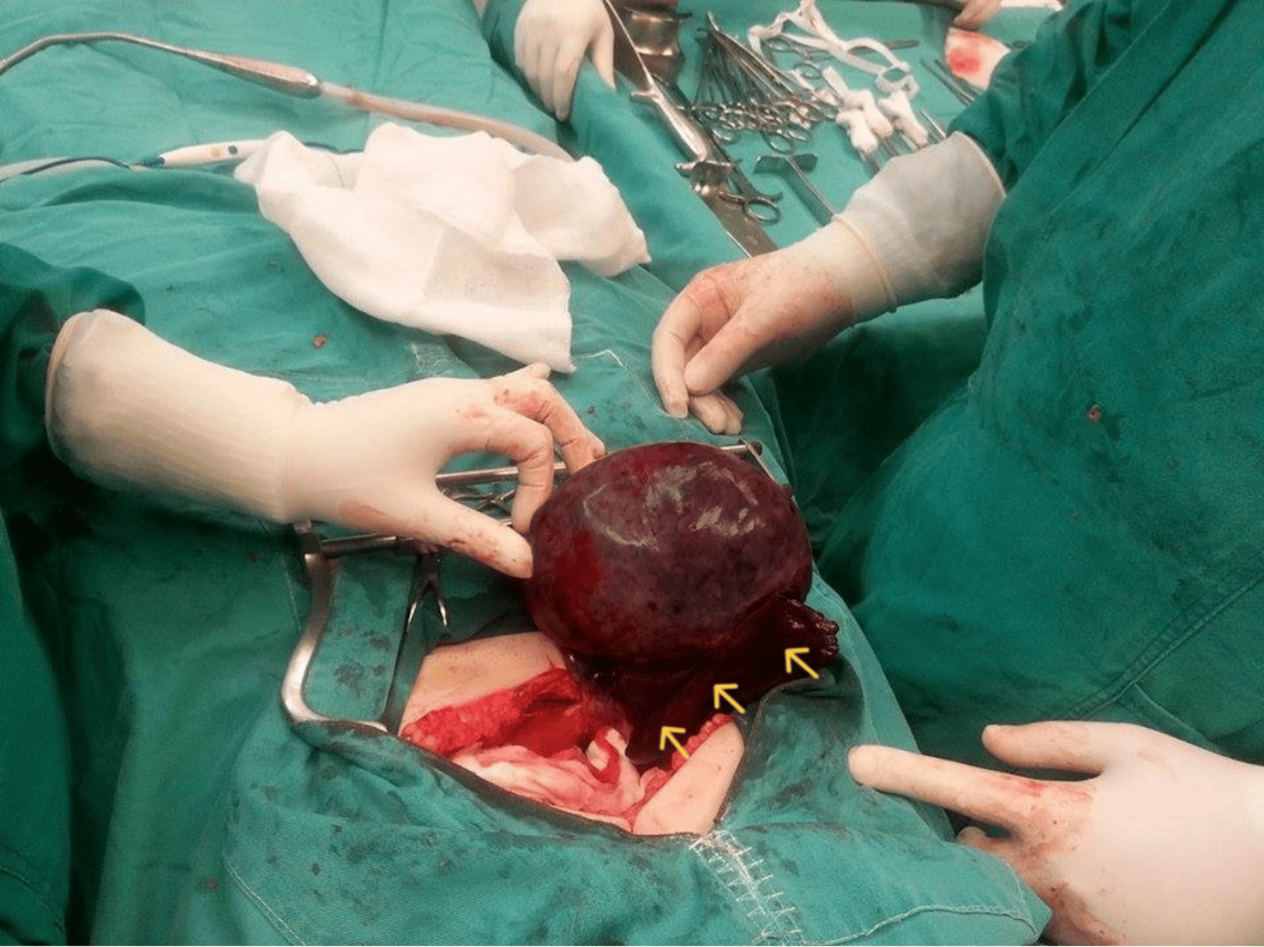
Intraoperative image of a torsed right adnexal mass with ovarian fibroma Ischemic and necrotic changes of the ovarian mass and the corresponding fallopian tube are clearly visible (yellow arrows)

Upon admission, intravenous antibiotics were started (Cefoxitin/Mefoxil® 2 g every six hours). The combined clinical, laboratory, and imaging findings prompted immediate exploratory laparotomy. Intraoperatively, a large solid ovarian mass (approximately 20 cm in maximum diameter) was found in torsion with clear signs of ischemia and necrosis, involving the right ovary and fallopian tube (Figure 2).

**Figure 3:**
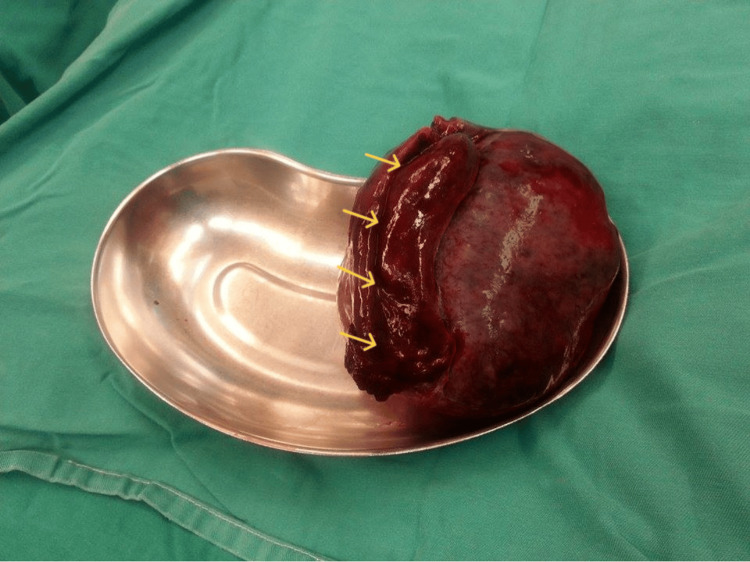
Surgical specimen of an ovarian fibroma with torsion The presence of a solid ovarian mass with necrotic lesions is evident, including involvement of the ipsilateral fallopian tube (yellow arrows)

**Figure 4:**
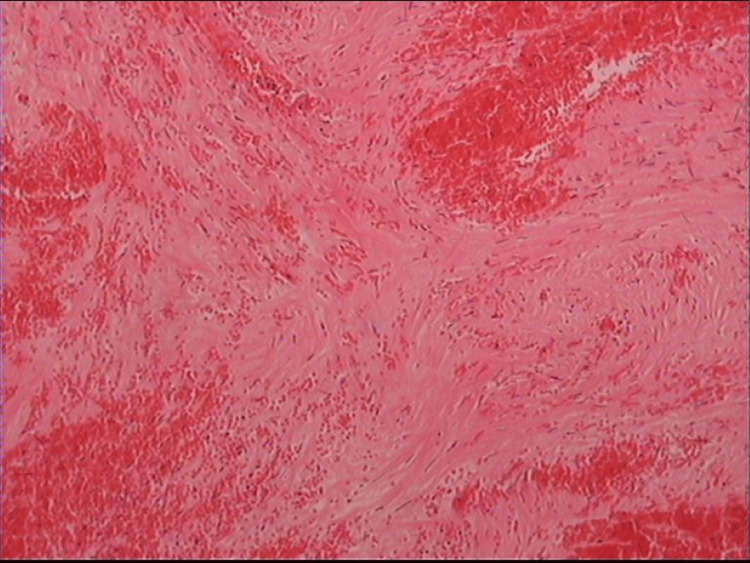
Histopathological image of an ovarian fibroma with torsion Vascular congestion and ischemic necrosis of the ovarian tumor support the diagnosis of torsion of the affected adnexa (magnification, x20)

Surgical untwisting of the ovary and the twisted vascular pedicle was performed. Observation of the adnexal mass after detorsion did not reveal any signs of reperfusion, suggesting viability of the right fallopian tube and associated ovary. The left adnexa appeared normal. A right salpingo-oophorectomy was performed. The surgical specimen (Figure 3) was sent for histopathological analysis, which confirmed the diagnosis of a torsed ovarian fibroma (Figure 4).

The early postoperative period was uneventful, and the patient was discharged on the fourth postoperative day. On the seventh postoperative day, a small fluid collection was noted at the left edge of the Pfannenstiel incision. Culture of the wound fluid showed no microbial growth. The aseptic wound infection was managed conservatively with cleaning every two days, achieving complete skin healing within 10 days.

## Discussion

The preoperative clinical diagnosis of torsed ovarian fibroma is challenging. Acute abdominal pain is the main clinical manifestation of torsion of an ovarian fibroma [8], often accompanied by nausea and repeated vomiting [6]. In rare cases, torsion-related acute abdominal pain may be associated with hemolytic anemia [9]. Uterine leiomyomas (especially those with cystic degeneration) and malignant ovarian neoplasms must be included in the differential diagnosis to avoid misdiagnosis, which can result in adverse outcomes and complications [10]. In our patient, the clinical picture was typical of acute adnexal torsion, with sudden, intense pain, multiple episodes of vomiting, and signs of peritoneal irritation. Nevertheless, the final diagnosis of a torsed ovarian fibroma was made intraoperatively and confirmed histologically.

Similar to the findings from the clinical examination, pelvic imaging also presents significant diagnostic challenges in the accurate preoperative diagnosis of torsed ovarian fibromas. Ultrasonography is considered the first-line imaging modality for the evaluation of ovarian torsion, which is usually associated with a benign cystic or solid mass, including ovarian fibroma. The presence of a large heterogeneous mass, located in the adnexal region within the affected ovary, which causes displacement of the uterus from the side of the affected ovary, is a common ultrasonographic finding in large torsed ovarian fibromas. Additionally, the ultrasonographic features of ovarian torsion include unilateral ovarian enlargement, the presence of free peritoneal fluid, and the absence of arterial or venous blood flow in the vascular pedicle of the affected adnexa, as demonstrated by color Doppler ultrasonography [11,12]. CT scan findings of torsion may include deviation of the uterus toward a well-defined, delayed-enhancing, heterogeneous solid mass; smooth thickening of the mass wall; thickening of the ipsilateral fallopian tube; presence of peripheral cystic structures; and ascites [12]. In our patient, neither ultrasound nor CT provided a definitive preoperative diagnosis.

The timely preoperative diagnosis of torsed ovarian fibromas is of paramount importance. Early recognition facilitates prompt surgical intervention, which may be critical in restoring adequate blood flow to the affected adnexa, thereby preventing irreversible functional damage to the ovary [12]. The management of ovarian torsion is also important for fertility preservation at reproductive age. According to the studies, providing detorsion within 24 h following ovarian torsion is related to a better ovarian reserve and minimizing ovarian tissue necrosis [13]. Intraoperative observation by the surgical team of the color of the ovary is a necessary step before making a decision to salvage ovarian tissue. Improvement in the color of the ovary and fallopian tube towards normal, approximately 20 minutes after diversion, indicates normal blood flow [14].

The treatment of choice for ovarian fibromas is surgical excision, performed either via an open or laparoscopic approach, followed by histopathological evaluation of the excised mass to establish a definitive diagnosis [15]. In young women, fertility-sparing surgery with excision of the fibroma while preserving healthy, functional ovarian tissue is recommended, particularly for those wishing to maintain future reproductive potential as part of family planning. In contrast, in older women, adnexectomy or total hysterectomy with bilateral salpingo-oophorectomy may be considered the preferred therapeutic approach [16,17]. In the case of our patient, who was only 17 years old, adnexal removal was deemed unavoidable. The extensive necrotic changes observed intraoperatively in both the ovarian mass and the corresponding fallopian tube, secondary to adnexal torsion (Figure 2 and Figure 3), precluded any surgical attempt to excise solely the ovarian fibroma while preserving the ovary and ipsilateral tube.

The prognosis of ovarian fibromas is excellent. Although the malignancy potential is low, surgical removal is necessary [18]. In cases of ovarian fibroma associated with Meigs syndrome, ascites and pleural effusion typically resolve after tumor excision [19].

## Conclusions

Torsion of an ovarian fibroma is a rare complication of ovarian fibromas. The development of a large ovarian fibroma followed by torsion is even more uncommon in adolescent patients, as illustrated in our case. Nevertheless, in young women presenting with acute abdominal pain accompanied by a pelvic mass, torsion of an ovarian fibroma should be included in the differential diagnosis to avoid adverse outcomes that may lead to further complications.
